# Genotype to phenotype mapping still needs underpinning by research in metabolism and enzymology

**DOI:** 10.1042/BSR20180520

**Published:** 2018-06-21

**Authors:** David A. Fell

**Affiliations:** Department of Biological and Medical Sciences, Oxford Brookes University, Headington, Oxford OX3 0BP, U.K.

**Keywords:** enzymology, genomics, metabolism

## Abstract

The article ‘Evidence that the metabolite repair enzyme NAD(P)HX epimerase has a moonlighting function’ by Niehaus et al. published in this issue illustrates a number of the problems that still arise when attempting to translate genotypes to phenotypes, such as for interpreting mutant phenotypes or building genome-scale metabolic models. In this case, the mutation concerned appears to map to an enzyme in one of the little-known but essential metabolite repair pathways that have been discovered in recent years. However, the bioinformatic and experimental evidence presented suggests that the annotated enzyme activity of the mutated gene product, whilst correct, accounts neither for the phenotype nor for the chromosomal and transcriptional associations of the gene. The bioinformatic and metabolomic evidence presented points to an additional but important role for the gene product in pyridoxal phosphate homoeostasis, thus adding the enzyme to the expanding list of those with a ‘moonlighting function’.

The article ‘Evidence that the metabolite repair enzyme NAD(P)HX epimerase has a moonlighting function’ by Niehaus et al. [1] in this issue could appear from its title to be just a footnote about a little-known metabolic pathway, but in fact it raises issues that impact on a number of very active areas of contemporary research. The question they raise is whether the enzyme NAD(P)HX epimerase (EC 5.1.99.6) has a second function outside the NAD(P)H repair pathway to which it is said to belong, related to pyridoxal 5-phosphate metabolism. In this case, the human metabolic disorder associated with mutation of the gene for the epimerase [2] might have other causes than accumulation of NAD(P)HX. Why should this investigation into a specific gene–protein–reaction association have implications outside the areas of metabolism directly involved? There are a number of reasons that interact to cause this.

One is that the accuracy of gene–protein–function assignments is central to the interpretation of organisms’ genome sequences and relating them to biological properties. The gene–protein–enzyme relationship was established by Tatum and Beadle [3] as the basis for the genetic encoding of metabolic pathways, so once complete genome sequences became available, there was the development of the technique of genome-scale metabolic modelling [4]. This takes the annotation of the genome sequence, identifies the metabolic enzymes and generates a list of the reactions they catalyse, which can then be used to compute metabolic properties, including the prediction of phenotypes of metabolic mutants. As more and more organisms are sequenced, this enables metabolic models to be built of organisms that have not been the subject of much biochemical investigation, so many of the gene–enzyme associations involved are indirect and derived by bioinformatic techniques such as sequence homology with known examples, and the enzyme–reaction associations from databases collating research literature. Though the reliability of these chains of inferences cannot be certain, there are other sources of uncertainty that need to be considered.

The paper alludes to one of these other sources of uncertainty: the completeness of our knowledge of metabolism, even in biochemically well-studied organisms. All genome sequences contain coding regions that cannot be assigned a function by bioinformatic methods, and it is likely that there are metabolic enzymes amongst these unannotated genes, especially as genome-scale metabolic models frequently have network gaps, where a reaction needed to ensure necessary connectedness of the network cannot be linked to a gene. However, in recent years there has been increasing awareness of new metabolic reactions associated with metabolite damage repair [5]. The repair pathways deal with compounds that are derived from metabolites, either through spontaneous chemical reactions (such as oxidation or hydrolysis) or through minor side reactions of enzymes with them because of structural similarities with natural substrates. Where these derived metabolites are not participants in normal central metabolism, they will accumulate unless either converted to a metabolite that is or excreted. This is the case in the paper by Niehaus et al. [1]: NAD(P)HX is formed spontaneously by the hydration of NADH and NADP, and the repair pathway involves returning it to its original state [6]. However, though the dehydratase component of the pathway is known to be necessary, it is uncertain whether the epimerase activity, which interconverts the R and S epimers (the substrate of the dehydratase), is needed given that the epimerization reaction occurs spontaneously. Indeed, part of the evidence in the paper is that inactivating the epimerase in *Escherichia coli* with a point mutation did not lead to abnormally high NAD(P)HX levels.

Another confounding issue in the mapping of genome sequence to a metabolic phenotype is the main topic of the paper: enzyme moonlighting, that is, an enzyme participating in another process within a cell that is distinct from its catalytic activity and often not even a direct metabolic function. There are now several hundred known examples [7]. The existence of moonlighting complicates the comparison with experimental data of gene essentiality computed from genome-scale metabolic models since the enzyme activity encoded by the gene may not be necessary for viability and functioning of the metabolic network, but the gene will be essential if the moonlighting function is. As in this paper, it also obscures whether the functional lesion in the case of a defective phenotype is associated with the metabolic process or the moonlighting function [8]. Niehaus et al. [1] demonstrate that, in the case of the epimerase, it is likely that it is the moonlighting function since the point mutation in *E. coli* did not have a significantly defective phenotype whereas deletion of the protein did. The nature of the defective phenotype pointed to a role for the epimerase protein in pyridoxal-associated metabolism, though whether that was via a different enzyme activity or some other process was not established.

In conclusion, Niehaus et al. [1] illustrate some of the consequences of the gaps in our knowledge of metabolism and enzymology for the exploitation of genome sequence information and for connecting genotype to phenotype. Though the rise of high-throughput techniques for the study of nucleic acids has eclipsed research in metabolism and enzymology, it is clear that continued progress the latter is still needed to allow full exploitation of the information in genome sequences. Consequences of these knowledge gaps have also been pointed out for synthetic biology and metabolic engineering developments [9] since the introduction of designed, novel enzymes and pathways into cells could create new damage pathways and metabolites in both the native and engineered metabolism that would need to be countered to achieve the desired outcomes.
